# Combining the DNA methylation markers of circulating tumor cells with immune infiltrating cells to assess recurrence and prognosis and to suggest a therapeutic strategy in stage III-IV colorectal cancer

**DOI:** 10.3389/fimmu.2025.1607548

**Published:** 2025-07-28

**Authors:** Juan Zhou, Danli Ye, Yuansen Li, Xuwen Lai, Wenzhi Cui, Wenyuan He, Ling Yu, Jingyi Wu, Guangning Yan, Chengyong Lei, Wei Wang

**Affiliations:** ^1^ Department of Oncology, General Hospital of Southern Theater Command, People’s Liberation Army of China, Guangzhou, China; ^2^ The First School of Clinical Medicine, Southern Medical University, Guangzhou, China; ^3^ Department of Pathology, General Hospital of Southern Theater Command, People’s Liberation Army of China, Guangzhou, China; ^4^ Graduate School, Guangzhou University of Chinese Medicine, Guangzhou, China; ^5^ Department of Urology, Nanfang Hospital, Southern Medical University, Guangzhou, China; ^6^ School of Basic Medicine, Southern Medical University, Guangzhou, China

**Keywords:** ctDNA methylation, CRC, prognosis prediction, tumor immune microenvironment, ZNF671, ZNF132

## Abstract

**Introduction:**

Circulating tumor DNA (ctDNA) methylation markers show potential for early detection of cancer metastasis. This study aimed to identify ctDNA methylation markers predictive of recurrence and prognosis in colorectal cancer (CRC) patients, and to explore the influence of the tumor immune microenvironment on outcomes.

**Methods:**

We analyzed 603 overlapping methylation markers from both plasma and tissue samples and developed a risk model to predict CRC recurrence and prognosis.

**Results:**

ZNF671 and ZNF132 were identified as key methylation markers. The model predicted relapse risk in stage III CRC patients with an AUC of 0.90 and prognosis in stage IV patients. High-risk patients exhibited a significantly higher early relapse rate (75.4% vs. 20%) and were more likely to have a low Immunoscore (IS), which correlates with poorer prognosis.

**Discussion:**

ZNF671 and ZNF132 methylation levels inversely correlate with Immunoscore and may serve as valuable biomarkers for CRC immunotherapy. These findings provide insights for improved prognostic evaluation and personalized treatment strategies.

## Introduction

Colorectal carcinoma (CRC) is the most common malignant tumor with frequent distant metastasis (1). Most of late-stage CRC metastasis sites are liver, while lungs and peritoneum metastasis are common as well. However, patients could still receive resection of the primary and metastatic lesions as long as the metastasis were limited, which may offer the only opportunity for potential cure or long-term survival (2–5). Stage III-IV CRC patients still has high recurrence rate, even if curative surgery and adjuvant chemotherapy was timely applicated (6). To date, despite the enormous efforts that the researchers have already put in, there are still little precise biomarkers to predict the recurrence risk or guide personalized treatment strategies.

Minimal residual disease (MRD) and circulating tumor cell (CTC) clusters are critical factors in cancer relapse and metastasis (7) but cannot be detected by routine imaging techniques (8–10). Research suggests that CTCs may undergo DNA methylation changes that facilitate metastatic seeding (7, 11). The epigenetic modification of DNA methylation has been shown to surpass somatic mutations in clinical applications, particularly in evaluating recurrence risk and developing personalized treatment strategies (12–15). Immunoscore could also predict the recurrence risk for CRC patients and be a predictive tool for cancer treatment (16, 17). Moreover, DNA methylation can interact with various immune cells and stromal components in tumor microenvironment (TME) which may potentially provide new avenues for cancer immunotherapy (18, 19). This study aims to identify novel methylation markers that could facilitate their clinical application and large-scale population screening. Furthermore, we investigate the prognostic significance of immune cell infiltration, as well as the relationship between the methylation model with the tumor immune microenvironment in CRC patients. The ctDNA methylation risk model, integrated with immune infiltrating cells in the TME, may provide valuable insights to predict and stratify CRC patients who are likely to benefit from adjuvant chemotherapy or immunotherapy.

## Methods

### Ethics approvals and consent to participate

The study was conducted in accordance with the Declaration of Helsinki and approved by the Ethics Committees of Southern Hospital (approval number: 2020-010) and General Hospital of Southern Theater Command (approval number: 2024GJJ036). All experimental plans were reviewed and approved by the committees before the clinical study began. Written informed consent was obtained from all participants prior to their enrollment in the study.

### Sample collection

Tissue and plasma samples from CRC patients were collected between May 2015 and October 2020 at the Southern Hospital of Southern Medical University and the General Hospital of the Southern Theater Command. The inclusion criteria were as follows: 1) pathologically and radiologically confirmed CRC diagnosis; 2) stage IV CRC patients who underwent liver, lung, or peritoneal metastasectomy with curative intent; 3) availability of tissue and/or blood samples for analysis. The exclusion criteria included: 1) a history of preoperative treatments; 2) incomplete clinical information; 3) a history of other cancers. Paired primary tumor and metastatic lesions from 27 stage IV CRC patients were analyzed to evaluate the correlation between primary tumor and its distant metastases. The healthy control group consisted of individuals with no significant medical history (such as cancer or chronic diseases) and aged over 18 years.

The sample collection followed our previous study protocol (20). In brief, a total of 139 tissue samples were collected, including 40 primary tumor samples, 42 metastatic stage IV tumor samples, and 57 normal mucosa samples from CRC patients. Additionally, 308 preoperative blood samples from CRC patients and 50 blood samples from healthy controls were included. Advanced CRC patients were regularly followed up after completing systemic treatment. During the first 3 years, imaging examinations were conducted every 6 months, followed by annual examinations until 5 years or the first radiological recurrence or death. Outcome data were collected through phone calls and outpatient records. Serum levels of CEA and CA199 had reference ranges of 0-5 μg/mL and 37 U/mL, respectively (21).

### Anchor Dx’s proprietary targeted methylation sequencing

All samples were processed using AnchorDx’s proprietary targeted methylation sequencing platform, which focuses on 12,624 cancer-specific CpG regions. Methylation sequencing was then performed using the Illumina HiSeqX Ten Sequencing System, with the detailed methods, including DNA extraction, bisulfite conversion, and construction of the AnchorIRIS™ pre-library, previously described in our published studies (13, 22, 23).

Data processing was performed using Illumina Sequencing Analysis Viewer and FastQC software to assess sequencing quality, and a custom algorithm was applied to trim low-quality bases. The sequencing reads were then aligned to the in silico converted hg19 reference genome using Bismark, and Picard was used to evaluate the sequencing performance, excluding CpG sites with coverage less than 30× or a missing rate greater than 0.20.

### Immunohistochemical detection and Immunoscore assay

The Immunoscore assay was conducted as previously reported (24). Briefly, experienced pathologists selected tumor blocks containing both the tumor and invasive margin from CRC patients. Sections of 4 μm were processed for standardized CD3 and CD8 immunohistochemical staining following the manufacturer’s instructions. Digital images of the stained tissue sections were captured at 20× magnification with a resolution of 0.45 µm/pixel. The densities of CD3 and CD8 in both regions of the whole slide were determined using dedicated Immunoscore software and then converted into percentiles by comparison with the Immunoscore database. The mean of the four percentiles was calculated and converted into an Immunoscore. The Immunoscore was typically categorized into three groups: 0%-25% (low), >25%-70% (intermediate), and >70%-100% (high). Immunohistochemical analysis and full scans of staining images were performed at Genecast Laboratory (Wuxi, China). The predictive accuracy of the Immunoscore was evaluated by the integrated area under the ROC curve, and the performance of risk prediction models was compared using the likelihood ratio test.

### ZNF671 and ZNF132 expression analysis and association with prognosis and the immune microenvironment

Statistical analysis was conducted using RStudio 4.4.1 with Bioconductor. Group differences were assessed using either Wilcoxon tests or paired t-tests, with a significance threshold set at p < 0.05. ROC curves were used to evaluate predictive accuracy, and Cox regression models were employed to analyze overall survival. The “ESTIMATE” package was used to calculate immune scores, while the “WGCNA” package identified gene co-expression modules. Additionally, the “xCell” package was utilized to examine the relationship between gene expression and immune cell infiltration.

### Statistical analysis

All statistical analyses and data visualizations were conducted using R (version 3.6.0) and Prism 8 (GraphPad Software). Pearson’s chi-square test and Fisher’s exact test were used to compare group differences. Survival data were analyzed with the Kaplan-Meier method, and Cox regression identified prognostic factors for recurrence-free survival (RFS). Hazard ratios (HR) and 95% confidence intervals (CI) were calculated using both univariate and multivariate Cox models. ROC curves were generated with the pROC R package (version 1.15.3) to evaluate the methylation model’s performance. LASSO and random forest methods were used for variable selection and risk model construction. Statistical significance was set at a p-value < 0.05.

## Results

### Patient characteristics and sample collection

**Figure 1:**
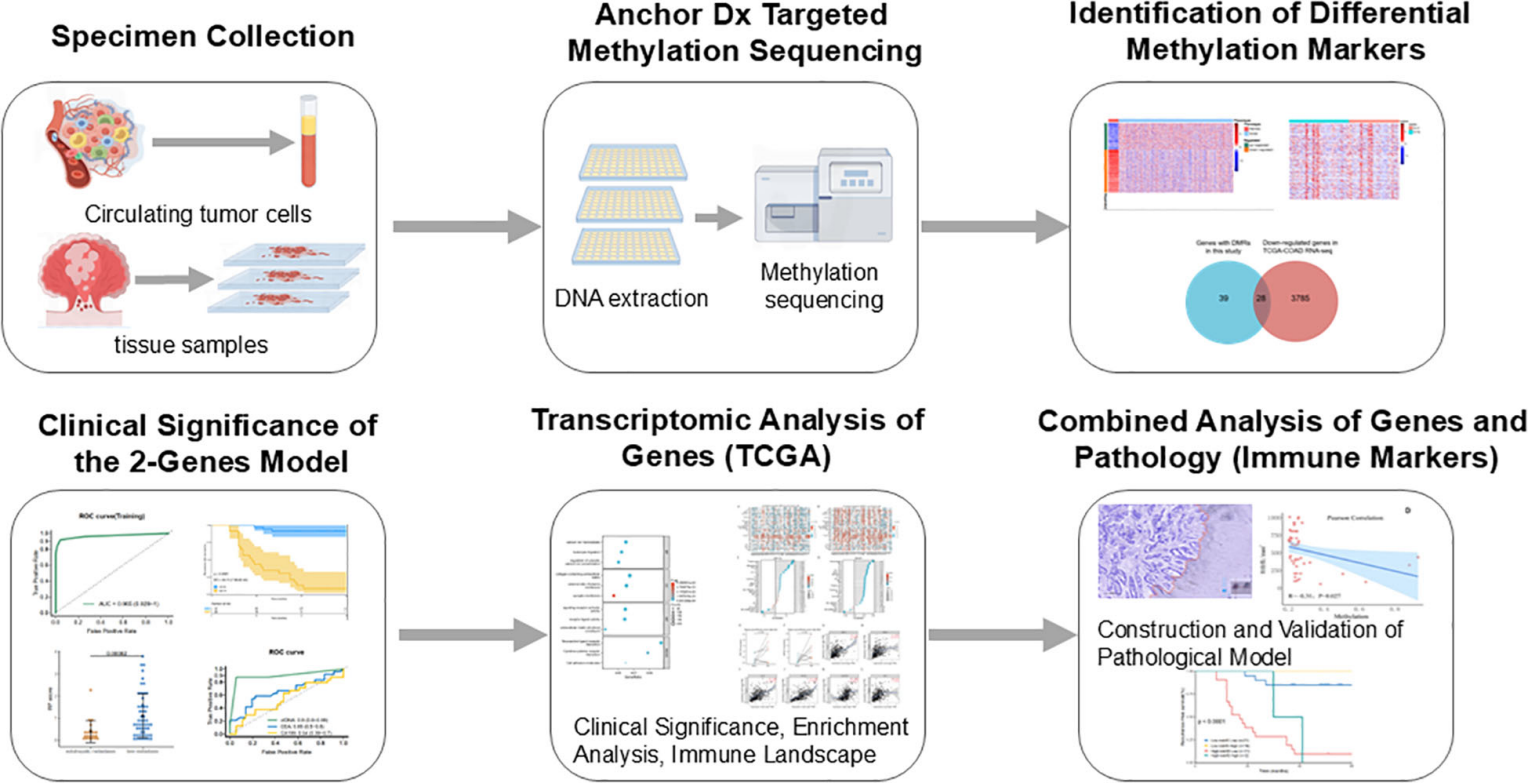
The design and process of our study are shown in the flow chart.

A total of 358 plasma samples (including 308 CRC patients and 50 healthy controls) and 139 formalin-fixed paraffin-embedded (FFPE) tissue samples (57 normal mucosa, 40 primary tumors, and 42 metastatic tissues from stage IV CRC patients) were collected in the study. Twelve tissue samples and 45 plasma samples were excluded due to DNA extraction quality control (QC) failure or low library yield. A total of 84 stage IV CRC patients underwent surgery, including 57 with liver metastases, 9 with lung metastases, and 18 with abdominal metastases. Detailed clinical characteristics are provided in **Supplementary Table S1**. Ultimately, 27 patients had matched preoperative plasma, primary tumor, and metastatic tumor tissue samples. The design and implementation of this study are outlined in **Figure 1**.

### Identification of differentially methylated markers from tissue and plasma samples in stage IV CRC patients

To identify potential methylation markers associated with distant metastasis in CRC, we first analyzed the differentially methylated regions (DMRs) between primary tumors and their metastatic tissues in stage IV CRC. However, few DMRs were detected between primary and metastatic tissues in stage IV disease. The methylation profiles of primary tumors were similar to those of distant metastatic tissues (**Supplementary Figures S1A, B**). A total of 2,274 DMRs were identified when we further analyzed DMRs between 53 normal mucosa samples and 74 stage IV tumor tissues (including 34 primary and 40 liver metastatic tissues). Differential methylation analysis of plasma samples revealed 981 DMRs that distinguished stage IV CRC patients from stage I/II populations. Finally, 603 DMRs were identified that were shared by CRC tissues and plasma. Nearly all of the CpG sites screened were hypermethylated in CRC tissues compared to normal mucosa, as confirmed in a previous study (22), The final set of 28 overlapping DMRs located within the promoter regions of down-regulated genes may reflect tumor burden in the peripheral blood of advanced CRC patients (**Supplementary Figures S1C, D**).

### Development and verification of a risk probability score for assessing tumor load

**Figure 2:**
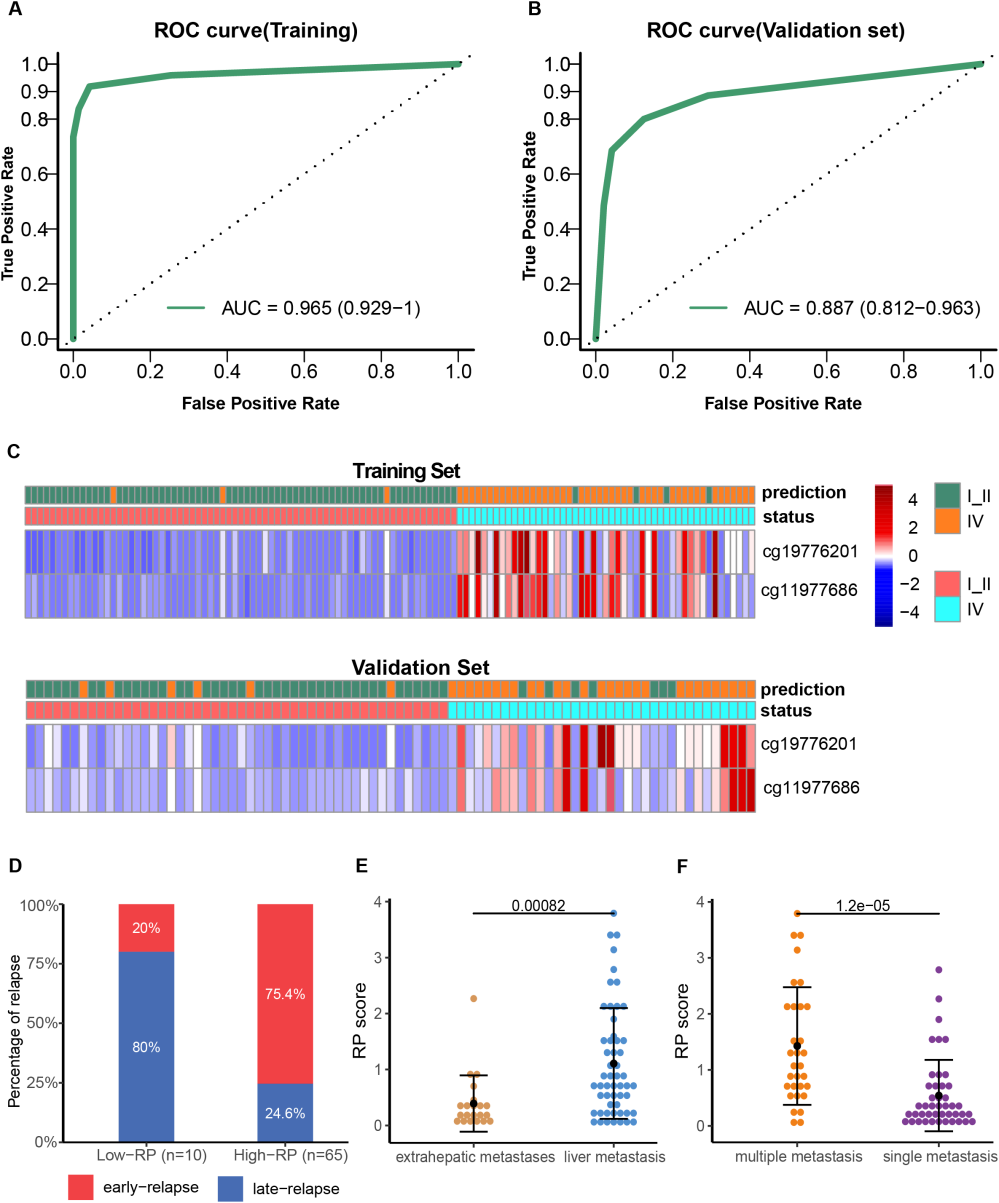
Development and validation of the two-methylation marker model. ROC curves of the risk model for late-stage CRC diagnosis in the training **(A)** and validation **(B)** cohorts. Heatmap showing the CpG markers between early-stage and late-stage IV CRC in the training **(C)** and validation cohorts. Each row represents an individual patient, and each column represents a CpG marker. The blue color represents a low methylation level, whereas the red color indicates a high methylation level. Comparison of early relapse rate for high and low RP score stage IV CRC patients **(D)**; RP scores difference between patients with liver metastasis and those with extrahepatic metastasis **(E)**; RP score difference between patients with multiple metastases and those with a single metastatic focus **(F)**.

Least Absolute Shrinkage and Selection Operator (LASSO) was applied for variable selection from CRC patients plasma samples. Training and validation cohorts at a 3:2 ratio were randomly assigned from 119 stage I/II and 84 stage IV samples. The DMRs with the highest classification Area Under Curve (AUC) were aligned based on the latest UCSC hg19 RefSeq annotation. A methylation risk assay model was constructed, which included cg11977686(ZNF671) and cg19776201(ZNF132), utilizing the random forest algorithm. This model was able to distinguish late-stage patients from early-stage patients in the training cohort, with a sensitivity of 91.84%, specificity of 95.78%, and an AUC score of 0.965 (**Figures 2A, C**, **Supplementary Table S2A**). Patients were classified into different groups based on these two methylation markers, and each patient was assigned a risk probability (RP) score derived from the random forest model.

The performance of the methylation marker assay in distinguishing stage IV from stage I-II patients was then evaluated in the validation cohort, achieving an AUC score of 0.887 (95% CI: 0.812–0.963), with sensitivity of 80.0% and specificity of 87.5% (**Figures 2B, D**, **Supplementary Table S2B**). Further, we divided the stage IV CRC patients with postoperative relapse into an early relapse group (within 12 months) and a late relapse group (after 12 months). Notably, the early relapse rate was significantly higher in patients with high RP scores (75.4%, 49 of 65 patients) compared to those with low RP scores (20%, 8 of 10 patients) (**Figure 2D**, OR: 12.25; 95% CI: 2.35–63.72; p=0.0012). Additionally, a significant difference was observed between patients with liver metastasis and those with extrahepatic metastases (P<0.001), with higher RP scores detected in patients with multiple metastases compared to those with a single metastatic focus (P<0.001, **Figures 2E, F**).

### The recurrence risk prediction for stage III CRC patients by the methylation risk assay

**Figure 3:**
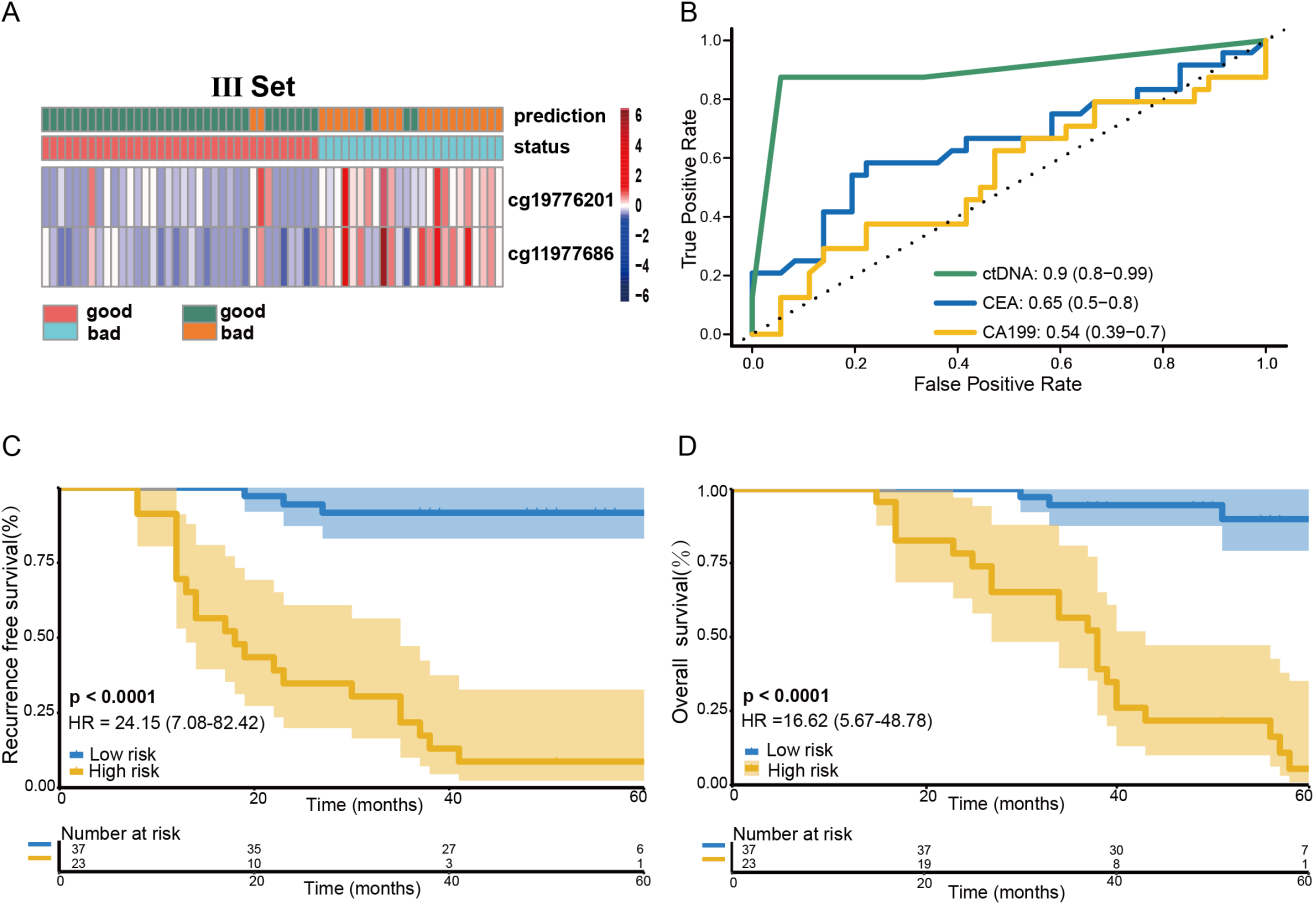
Predictive value of the RP score model on the prognosis of stage III CRC patients. Heatmap showing the methylation markers ZNF132 and ZNF671 in stage III CRC patients **(A)**. The methylation risk assay demonstrated superior performance in predicting recurrence risk compared to preoperative CEA and CA199 levels for stage III CRC patients **(B)**. Patients in the low-risk RP score group exhibited a significantly higher RFS **(C)** and OS **(D)** compared to those in the high-risk RP score group.

The performance of the methylation risk model in monitoring recurrence risk was further evaluated in a cohort of 60 stage III CRC patients (**Figure 3A**). ROC analysis revealed that the risk model outperformed traditional clinical markers, such as preoperative CEA and CA199 levels (**Figure 3B**). Based on the RP score calculated from the methylation assay, 24 patients were identified as being at high recurrence risk. After a median follow-up of 79.3 months (range: 57-112 months), 21 of these high-risk patients developed liver recurrence and/or distant metastasis (87.5%, 95% CI: 67.6-97.3%; **Table 1**). In contrast, recurrence occurred in only 2 out of 36 low-risk group patients (5.6%, 95% CI: 0.70-18.7%). The recurrence risk at 5 years was significantly higher in the high-risk group compared to the low-risk group (**Figure 3C**, HR: 24.15; 95% CI: 7.08-82.42, P<0.0001). Furthermore, patients in the low RP score group had a significantly higher overall survival (OS) rate compared to patients in the high RP score group (**Figure 3D**, HR: 16.52; 95% CI: 5.67-48.78, P<0.0001).

**Table 1:** Confusion matrices built from the ctDNA-based methylation marker prediction in stage III CRC patients.

Stage III cohort	Real progression	Real stability
Predicted progression	21	2
Predicted stability	3	34
Sensitivity (%)	87.5 (95% CI: 67.6-97.3)	
Specificity (%)		94.4 (95% CI: 81.3-99.3)

Univariate analyses showed that both the methylation risk model and preoperative CEA levels were significantly associated with 5-year RFS) in stage III CRC patients. Notably, preoperative methylation of ZNF132 and ZNF671 was strongly associated with 5-year RFS and emerged as an independent risk factor for recurrence in multivariate analysis (HR: 78.94; 95% CI: 14.53-428.85; P=4.21e-07, **Supplementary Table S3**).

### The association analysis of ZNF671 and ZNF132 gene expression with clinical prognostic factors

We further validated the reliability of the model by analyzing the differential expression of ZNF671 and ZNF132 in common tumors and adjacent normal tissues from the TCGA database. The results showed that the promoter methylation levels of ZNF671 and ZNF132 were significantly higher in CRC tumor tissues compared to normal mucosa (**Supplementary Figures S2A, B**). ROC analysis revealed that both ZNF671 and ZNF132 exhibited high diagnostic accuracy for CRC, with AUC values of 0.917 and 0.889, respectively (**Supplementary Figure S2C**).

Survival analysis indicated that low expression of ZNF132 (which corresponds to high methylation) was associated with significantly poorer survival in CRC patients compared to high expression (P < 0.05) (**Supplementary Figure S2D**). However, no significant survival difference was observed for ZNF671 (P > 0.05).

To further explore the potential prognostic roles and pathways of ZNF671 and ZNF132, we conducted Gene Ontology (GO) and Kyoto Encyclopedia of Genes and Genomes (KEGG) enrichment analyses (**Supplementary Figures S2E, F**). The enriched pathways included signal transduction, cell adhesion, immune regulation, metabolism, and disease development, underscoring the roles of ZNF671 and ZNF132 in tumor progression and prognosis.

### ZNF132 and ZNF671 expression involved in regulating immune and may suggest a therapeutic strategy in CRC patients

**Figure 4:**
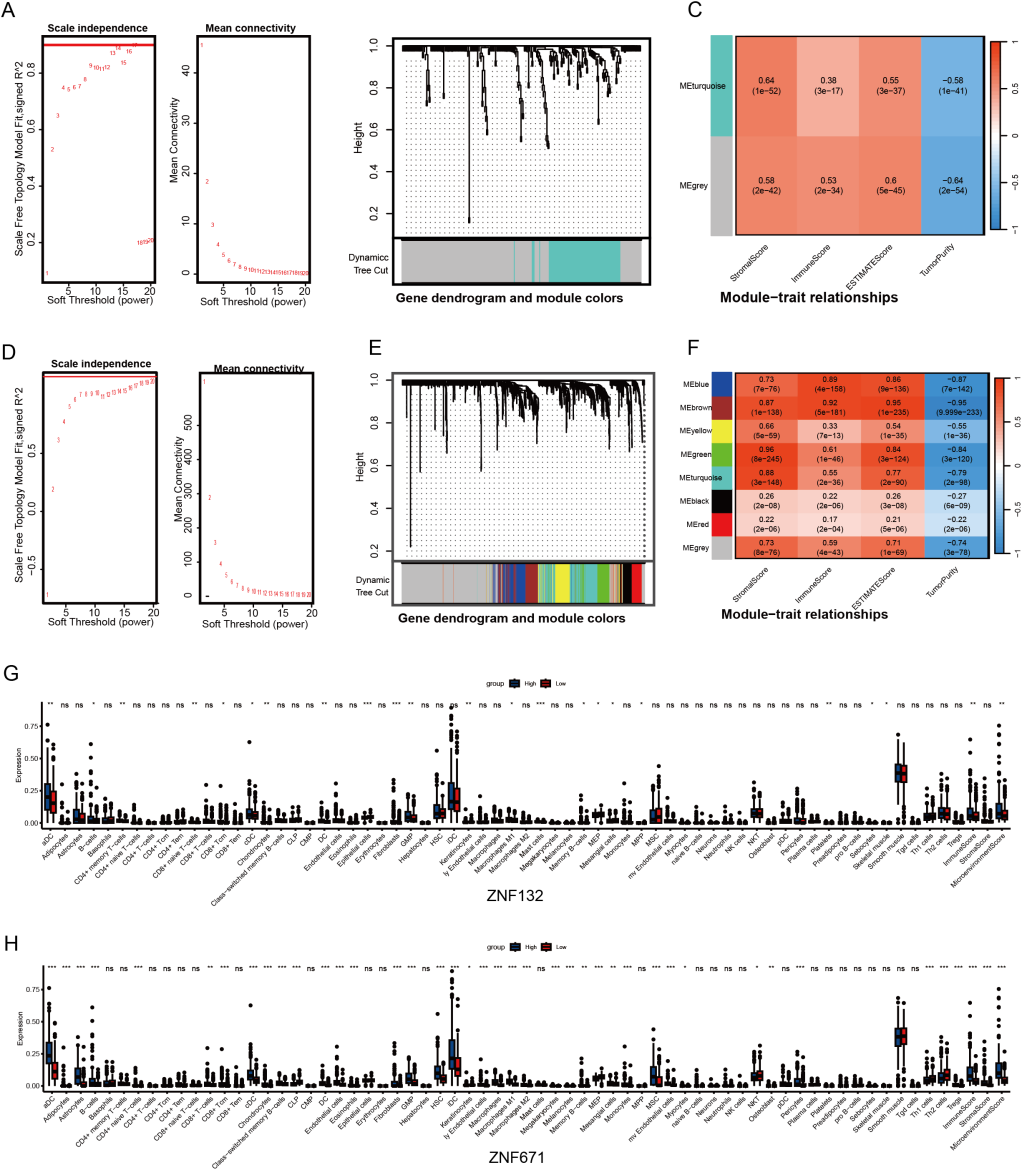
Association analysis of ZNF132 and ZNF671 expression with the immune microenvironment of CRC. Analysis of network topology for various soft-thresholding powers, determining the optimal power for constructing the ZNF132-associated scale-free network **(A)**. Gene cluster tree, showing the module division of ZNF132-associated genes **(B)**. Module-trait associations, illustrating the relationship between ZNF132-associated modules and immune infiltration **(C)**. ZNF671 **(D-F)**. ZNF132 **(G)** and ZNF671 **(H)** expression levels in relation to immune cell infiltration in general and the abundance of specific cell types *p < 0.05, **p < 0.01, ****p < 0.001.

Given the significant enrichment of ZNF132 and ZNF671 in immune function and related pathways, we used immune scores from the ESTIMATE algorithm as phenotypic data and applied WGCNA to identify gene modules significantly associated with ZNF132 and ZNF671 expression (**Figures 4A–F**). The identified modules suggest a potential link between ZNF132 and ZNF671 expression and immune cell infiltration, as well as immune-related pathways, highlighting their involvement in regulating immune responses and interacting with stromal cells.

Analysis with the xCell package revealed that ZNF132 is primarily associated with the immune infiltration of epithelial cells, fibroblasts, and Granulocyte-Monocyte Progenitor (GMP) cells (**Figure 4G**). In contrast, ZNF671 exhibited significant correlations with the infiltration of most immune and stromal cell types (**Figure 4H**). In summary, both ZNF132 and ZNF671 may play crucial roles in regulating immune responses and tumor-stroma interactions.

**Figure 5:**
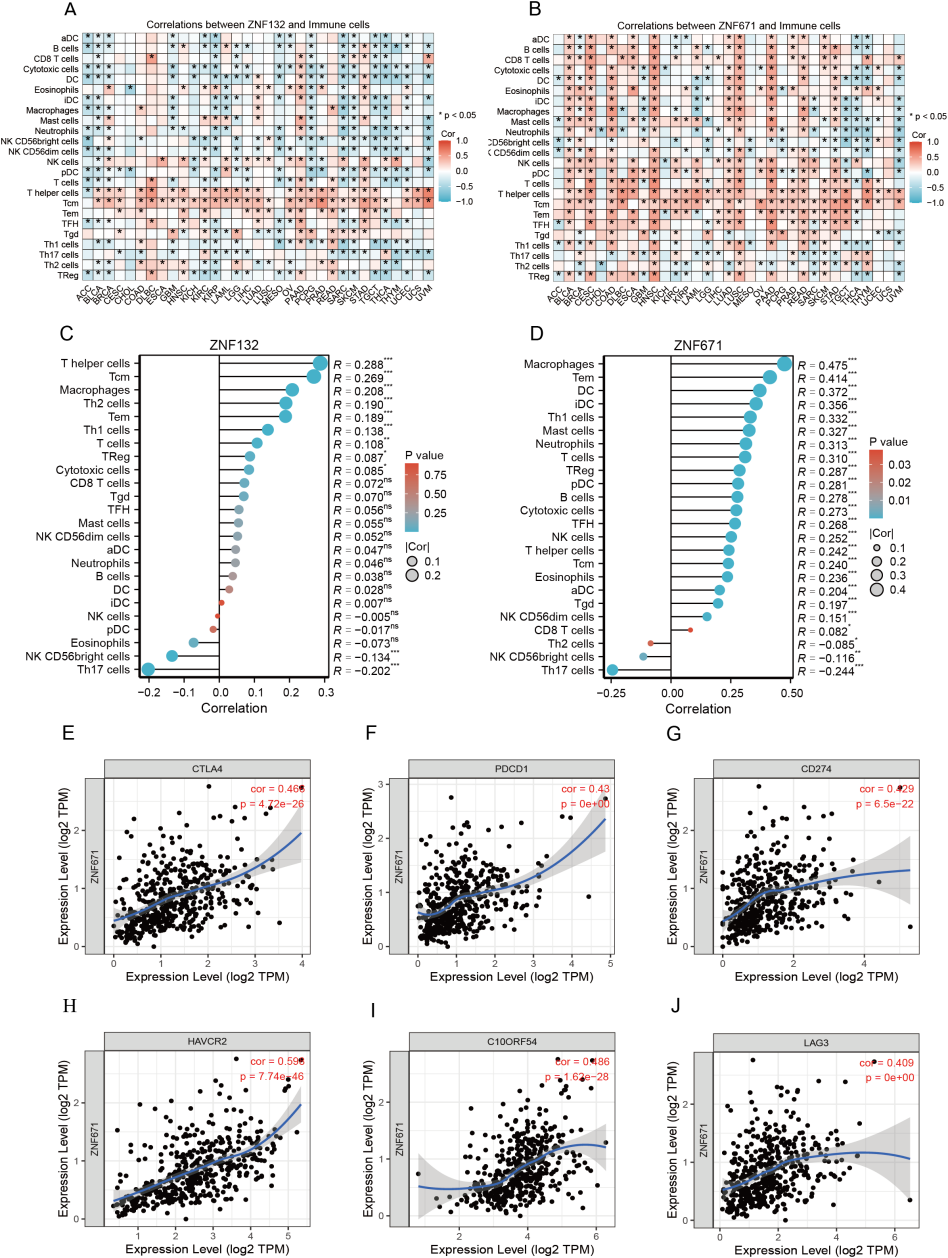
Correlation of ZNF132 and ZNF671 with immune cells and immune checkpoint genes, and changes after chemotherapy. Correlation of ZNF132 **(A)** and ZNF671 **(B)** expression with various immune cells in different cancer types. Correlation of ZNF132 **(C)** and **(D)** ZNF671 expression with various immune cells in CRC. Correlation of CTLA4 **(E)**, PDCD1 **(F)**, CD274 **(G)**, HAVCR2 **(H)**, C10ORF54 **(I)**, and LAG3 **(J)** expression with ZNF671 in CRC.

Further analysis across various cancers showed that ZNF132 and ZNF671 expression was significantly correlated with immune cell infiltration (**Figures 5A, B**). In CRC, ZNF132 primarily correlated with T helper cells (r=0.31) and T central memory (Tcm) cells (r=0.30) (**Figure 5C**). Meanwhile, ZNF671 showed the strongest correlation with macrophages (r = 0.5), followed by T effector memory (Tem) cells (r=0.42) (**Figure 5D**). Additionally, ZNF671 expression was moderately correlated with key immune checkpoint genes, including PD-1, PD-L1, and CTLA-4, in CRC (**Figures 5E–J**).

### Prognostic value of the Immunoscore in stage III CRC patients and its correlation with the risk model

**Figure 6:**
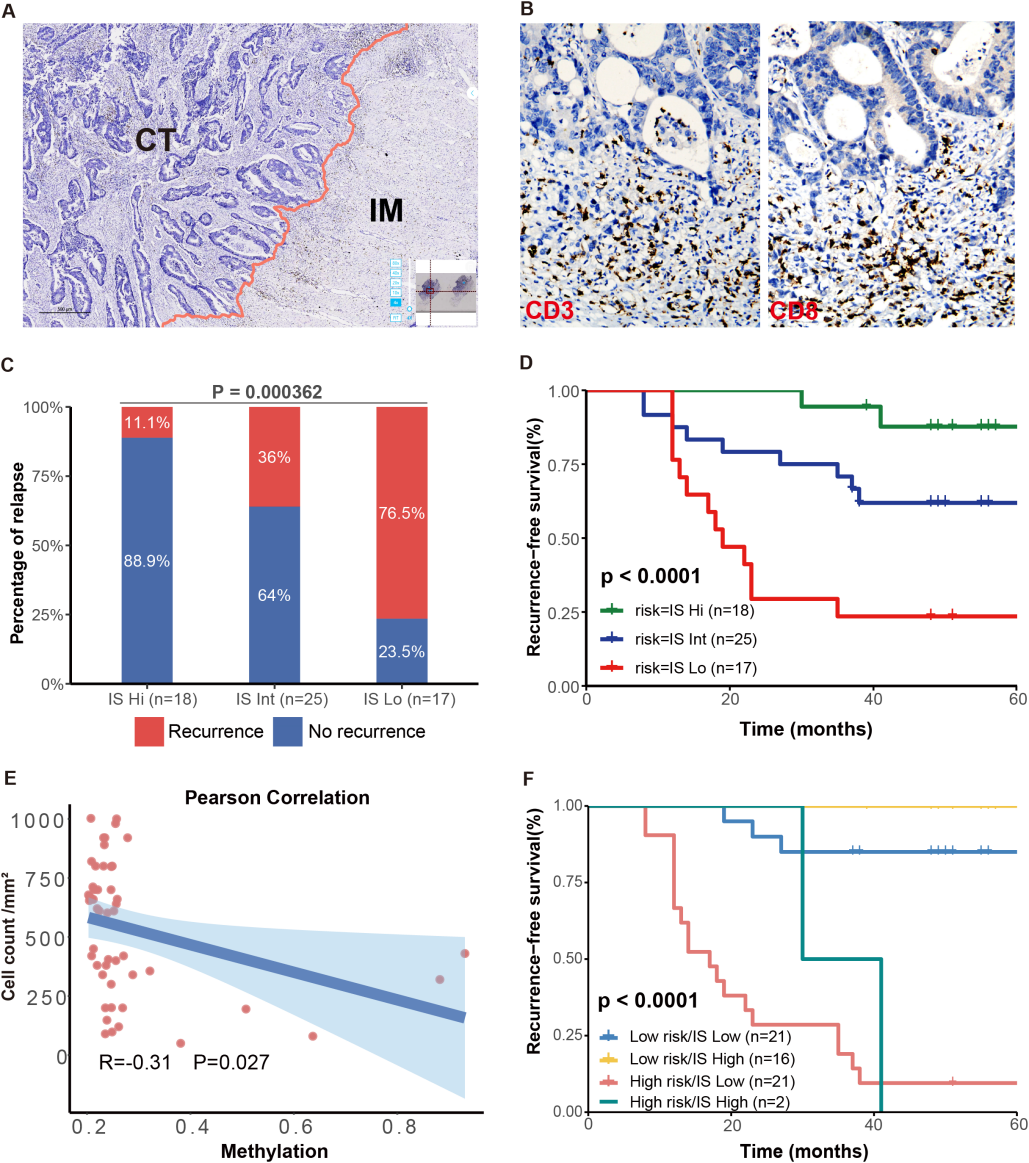
Prognostic significance of IS and association with the methylation risk model. Schematic diagram illustrating the separation of tumor regions into the central tumor (CT) and invasive margin (IM) regions **(A)**. Immunohistochemical staining of CD3 and CD8 in CRC tissues **(B)**. The 5-year RFS were significantly different among patients with high, intermediate, and low IS **(C)**. High IS CRC patients had significantly better 5-year RFS compared to patients with IS-Int or IS-low **(D)**. IS was negatively correlated with the RP score **(E)**. Kaplan-Meier curves for RFS in patients with different risk and IS levels **(F)**.

Building on our previous analysis, which revealed that the expression levels of ZNF132 and ZNF671 were associated with the enrichment of immune-related pathways, we assessed immune scores in a matched cohort of 60 stage III CRC patients. The immune scores were categorized into high, intermediate (Int), and low groups, with 18 (30%), 25 (41.7%), and 17 (28.3%) patients, respectively (**Figures 6A, B**). The 5-year recurrence rates for IS-high, IS-Int, and IS-low patients were 11.1%, 36%, and 76.5%, respectively (P < 0.001) (**Figure 6C**). Notably, CRC patients with high immune scores exhibited significantly better 5-year RFS compared to those with intermediate or low immune scores (**Figure 6D**).

Additionally, a negative correlation was observed between the immune score and methylation risk score (R = -0.31, P = 0.027, **Figure 6E**). This finding aligns with previous analyses from the TCGA database, where CRC patients with lower methylation levels of ZNF132 and ZNF671 generally exhibited more lymphocytes infiltration. The combination of high immune scores and low methylation risk scores provided the best prognostic prediction (**Figure 6F**). These results suggest that the combined assessment of immune score and methylation risk score holds significant clinical prognostic and therapeutic value.

### Assistance of the methylation model in the tumor staging of CRC patients

Interestingly, four CRC patients were initially suspected of having liver or lung metastasis based on imaging examination at the time of first diagnosis. Among these, two patients had elevated CEA levels, while the other two had normal CEA levels. However, all four patients were classified into the low recurrence risk group by the methylation assay model. After an average follow-up of 79.3 months, none of these patients experienced tumor progression (**Supplementary Figure S3**). Further investigation revealed that the suspicious pulmonary or intrahepatic lesions were actually old tuberculosis or hepatic hemangioma.

This finding underscores that, in some advanced-stage CRC patients, imaging examinations alone can be ambiguous and may not accurately determine the tumor stage. The methylation risk model, however, may provide valuable assistance in determining the TNM staging and predicting the outcomes for such equivocal cases.

## Discussion

Plasma circulating tumor DNA (ctDNA) has emerged as a promising non-invasive tool for the repeated evaluation of tumor burden and epigenetic profiles. Cancer development is closely associated with epigenetic alterations (25). Aberrant DNA methylation plays an important role in many solid tumors and has been extensively studied as a promising marker for early diagnosis and prognostic evaluation across various tumor types (12–15, 26, 27). In patients with stage III colon cancer, adjuvant chemotherapy prevents recurrence and tumor progression by eradicating minimal residual disease or clusters of tumor cells hidden in the peripheral blood (28, 29). However, limited clinical success has been achieved in identifying patients at high risk of recurrence after surgery or completion of standard adjuvant treatment. For late-stage CRC patients with distant metastatic disease, 70% will experience recurrence within 2 years, despite some being cured following liver resection (6, 30). Identifying late-stage CRC patients with a high risk of rapid progression could help personalize treatment strategies and improve patients’ outcomes. The primary factors underlying cancer relapse and metastasis are minimal residual disease or clusters of tumor cells circulating in the peripheral blood. Postsurgical ctDNA analysis can detect minimal residual disease and recurrence in CRC, while preoperative ctDNA detection is associated with tumor burden and worse disease-specific survival (DSS) (8, 31–33). Preoperative ctDNA methylation levels have been shown to enable prognosis prediction in various cancers, including CRC, hepatocellular carcinoma, and lung cancer, due to the abundant methylated loci in both cancer tissues and cfDNA (12–14, 34).

Due to advances in next-generation sequencing (NGS) technology, aberrant methylation specific to CRC has been identified, facilitating the screening of ctDNA methylation biomarkers for early detection and prognosis prediction of CRC (14, 20, 34). However, most of the established models include too many markers and rely on NGS assays, which involve high economic and time costs, thereby limiting their clinical application and large-scale screening. In this study, based on an increased sample size and improved data analysis, we developed a new methylation model that only includes methylation markers ZNF132 and ZNF671. This model demonstrated diagnostic performance comparable to previous reports, achieving an accuracy of 87.5%. Kaplan-Meier survival analysis revealed that the high-risk group, as determined by the RP score, had significantly lower survival rates compared to the low-risk group. Additionally, for stage IV CRC patients who underwent resection of all visible disease, rapid progression was significantly higher in the high-risk group (91.4%) compared to the low-risk group (13.3%) (p < 0.001), a prediction not addressed in other studies previously.

High methylation levels typically lead to the silencing of tumor suppressor genes. Analyzing the methylation levels of ZNF132 and ZNF671 and their impact on gene expression in CRC patient samples helps clarify the roles of these biomarkers in tumor development and progression. Based on the TCGA database, we found that high methylation of ZNF132 and ZNF671 results in gene silencing or reduced expression, which in turn influences the tumor progression and correlates with the prognosis of CRC patients. Gene enrichment analysis of tumor samples with high ZNF132 and ZNF671 methylation revealed their involvement in immune pathways and cell proliferation, highlighting their biological functions and potential prognostic value.

A previous study proposed that the intra-tumoral immune contexture could be used to evaluate the clinical outcomes of solid tumors (35). Galon et al. summarized and calculated the density of CD3+ and CD8+ T cells within the tumor and its invasive margin to define the Immunoscore (IS), which provides a reliable estimate of the risk of recurrence in CRC patients (24). Our study of 60 stage III CRC patients also confirmed that the IS is a reliable predictor of recurrence risk in CRC. High IS was associated with the lower risk of recurrence and the highest RFS and overall survival (OS). Furthermore, we found a negative correlation between methylation levels and IS. Specifically, patients with high ZNF132 and ZNF671 methylation levels generally exhibited lower immune cells infiltration. Further analysis using WGCNA and xCell to explore the correlation between ZNF132 and ZNF671 expression and immune cell infiltration in the TME indicated that both genes are involved in regulating immune responses and tumor-stroma interactions. ZNF671 expression showed the strongest correlation with macrophages and effector memory T cells (Tem), a moderate correlation with key immune checkpoint genes (PD-1, PD-L1, CTLA-4, etc.) in CRC. ZNF132 exhibited weak correlations with T helper cells and central memory T cells (Tcm). Previous research has shown that ZNF671 inhibits CRC progression by suppressing the Notch signaling pathway (36), which is critically involved in immune cell development and function, including T-cell differentiation (37), macrophage polarization (38), and regulatory T cell maintenance (37). Given the established role of Notch signaling in immune regulation, it is plausible that ZNF671 may modulate the tumor immune microenvironment through this pathway. However, the mechanism of ZNF132 in immune regulation remains largely unexplored. Future studies incorporating mechanistic assays and immune cell functional experiments will be essential to clarify the roles of ZNF132 and ZNF671 in CRC immunity. In addition to the epigenetic and immune-related markers discussed above, other inflammatory or metabolic biomarkers may also offer prognostic value. For example, urinary 11-dehydrothromboxane B2 (11-dehydroTXB_2_), a stable metabolite of thromboxane A2, has been implicated in tumor-associated inflammation, immunosuppression, and metastasis (39–41). Its measurement may provide non-invasive insight into the systemic immune-inflammatory status of CRC patients.

Finally, an interesting finding in our research is that the methylation risk model can assist in TMN staging of CRC to some extent. For suspicious liver or pulmonary nodules detected at the time of primary cancer diagnosis, a low methylation risk score could help refine CRC tumor staging, potentially avoiding misdiagnosis and excessive treatment.

Overall, we developed a more concise two-methylation-marker model. This model could evaluate the relapse risk of stage III CRC patients and predict the prognosis of late-stage patients. Database analysis further confirms that ZNF132 and ZNF671 play significantly diagnostic and prognostic roles in CRC. Both genes are linked to immune cell infiltration, immune-related pathways, and show a strong correlation with the immune stroma, highlighting their potential value as predictive markers for immunotherapy. Furthermore, our study provides further confirmation that the IS can predict the prognosis of stage III CRC patients and is negatively correlated with the methylation risk model. Previous studies have strongly suggested higher sensitivity to immunotherapy in low-risk Cutaneous Melanoma patients (42). Other studies have also shown a reduced response to anti-PD-L1 therapy in lung adenocarcinoma patients when the risk score is high (43, 44). Whether the IS and methylation risk model could offer a new categorization to guide therapeutic strategy in CRC patients needs to be tested in future studies. Our findings could improve management strategies and facilitate early intervention and personalized adjuvant therapies for CRC patients.

## Limitations

There are several potential limitations to our study. First, the plasma samples, which included 119 cases of early-stage CRC and 84 cases of late-stage CRC, were randomly divided into training and validation sets at a 3:2 ratio. This resulted in a relatively small number of samples in the validation set (83 cases). As a result, the robustness of the methylation model should be further validated with a larger sample size.

## Data Availability

The data supporting the findings of this study have been publicly deposited on GitHub at: https://github.com/zxcwuzheng/stopCOAD. This repository contains the complete DNA methylation beta value dataset and grouping information, along with detailed data documentation. All data comply with ethical standards and contain no personally identifiable information. Some datasets presented in this article are not readily available due to the ethical considerations and intellectual property regulations that govern the sharing of clinical data and plasma assay data. Requests to access these datasets should be directed to the corresponding author.
