# Supplementary material for: Combining the DNA methylation markers of circulating tumor cells with immune infiltrating cells to assess recurrence and prognosis and to suggest a therapeutic strategy in stage III-IV colorectal cancer
Source: Front Immunol. 2025 Jul 28;16:1607548. doi: 10.3389/fimmu.2025.1607548 (PMC12336224; doi:10.3389/fimmu.2025.1607548)
Supplement: Supplementary file 1 [file DataSheet1.docx]

**Supplementary Materials for**

**Combining the DNA methylation markers of circulating tumor cells with immune infiltrating cells to assess recurrence and prognosis and to suggest a therapeutic strategy in stage III-IV colorectal cancer** Wang et al.

**Figure Legends**

**Supplementary Figure 1. Identification of differentially methylated markers specific to CRC**

**
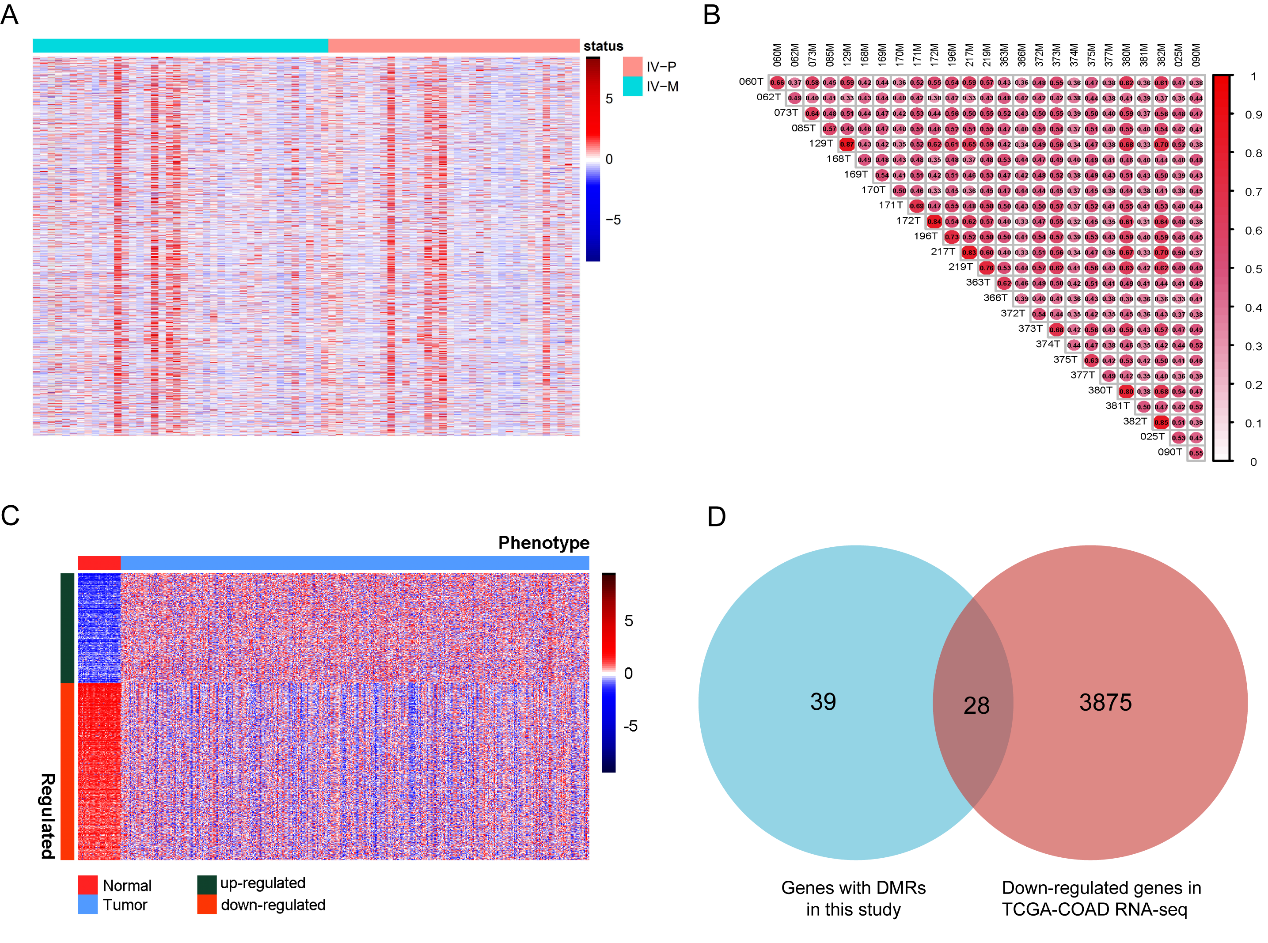
**

No significant DMRs were identified between primary and metastatic tissues from stage IV disease (A). Correlation bubble chart of 27 primary tumor and matched metastatic loci (B). The size of the colored bubbles represents the strength of correlation. Red: Positive correlation; Blue: Negative correlation. The bigger and darker the bubble is, the stronger the correlation is. Transcriptome profile of DEGs from TCGA-COAD (C). 28 overlapping DMRs between the anchored differential genes and the DEGs from TCGA-COAD by RNA-seq (D).

**Supplementary Figure 2 Analysis of ZNF132 and ZNF671 methylation, diagnostic performance, prognostic impact, and related biological pathways in CRC from the TCGA database.**


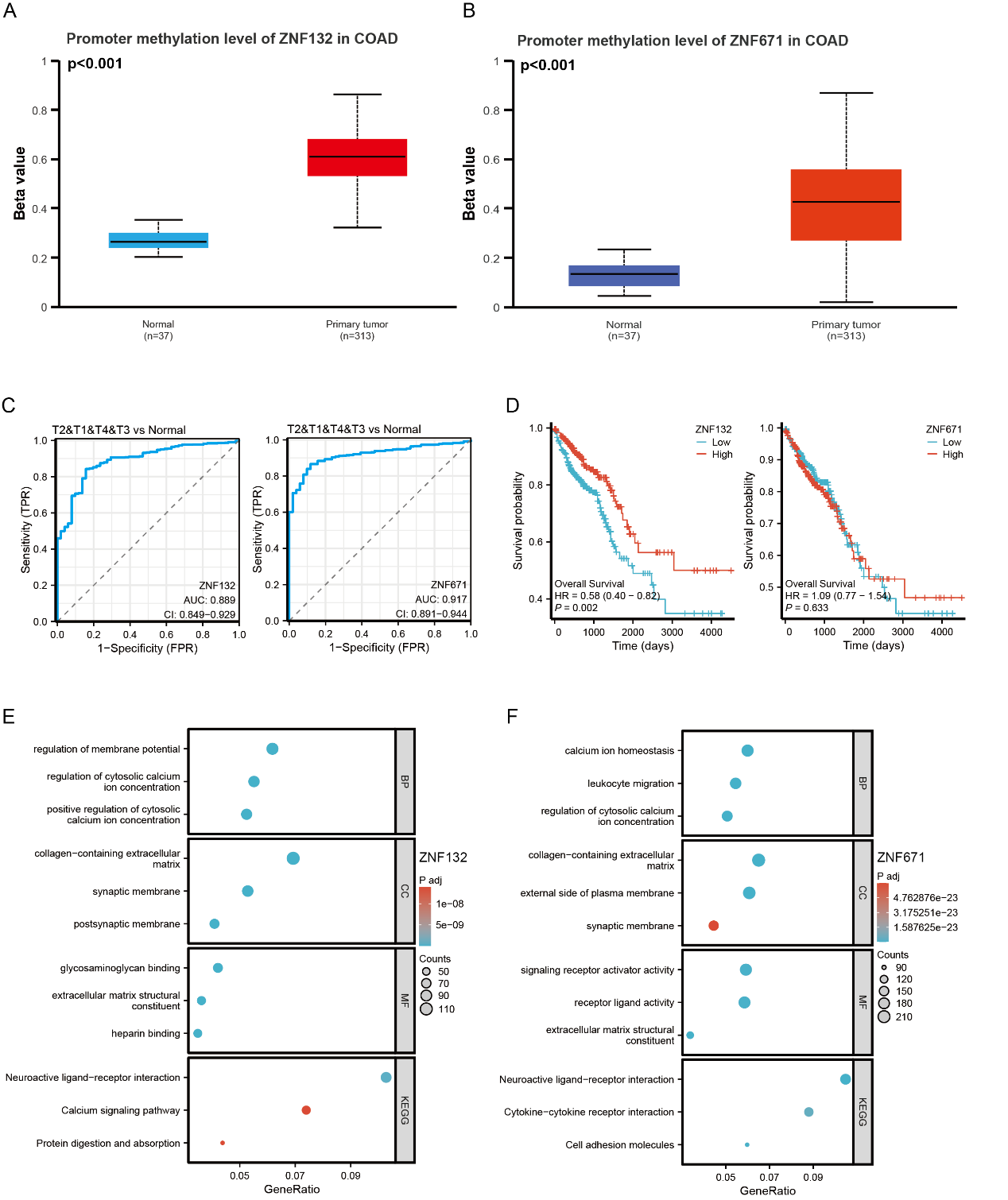


ZNF132 (A) and ZNF671 (B) methylation differernce between CRC tumors and normal tissues. ROC analysis of ZNF671 and ZNF132's diagnostic performance in CRC patients (C). Cox regression analysis of ZNF671 and ZNF132's association with patient survival (D). Enrichment analysis identifying relevant biological processes and signaling pathways (E)(F) .

**Supplementary Figure 3. Assistance of the methylation model in the tumor staging of CRC patients.**

**
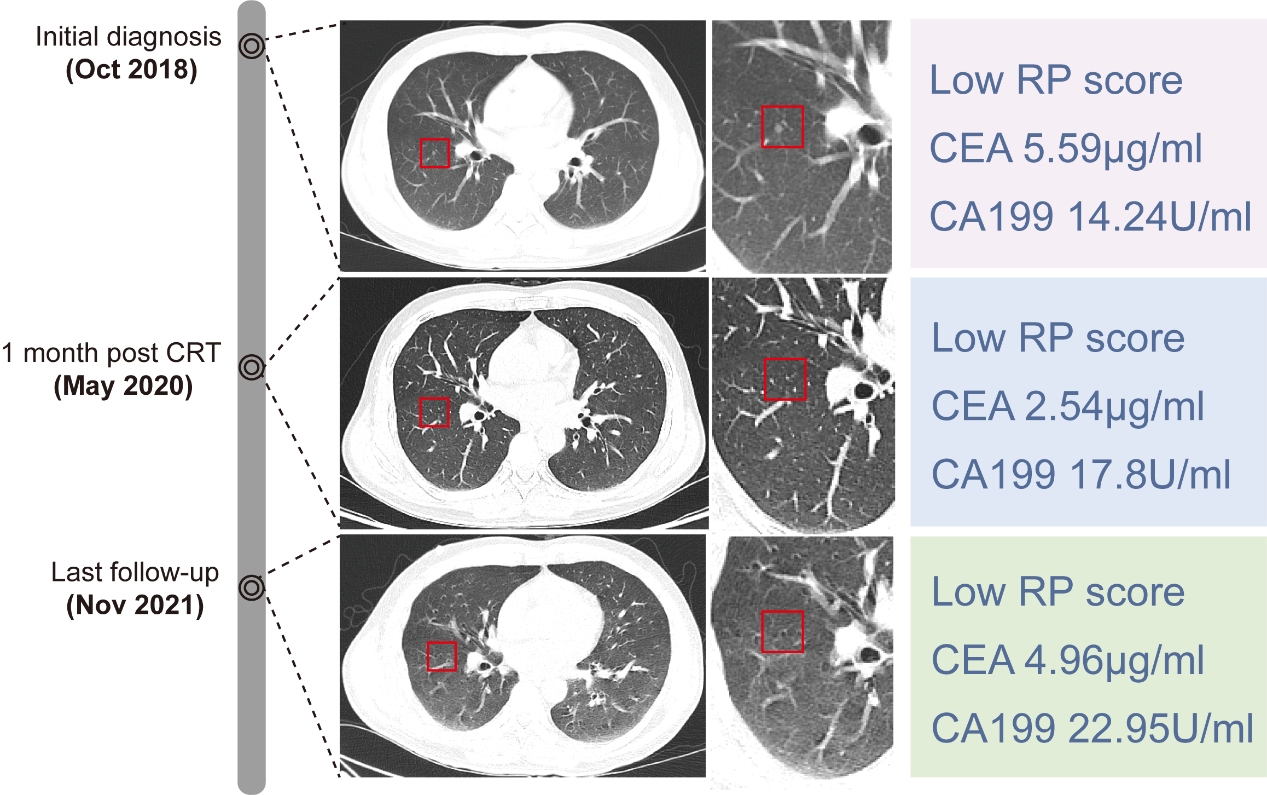
**

The methylation risk model help to determine the TMN staging for those equivocal CRC patients in imaging examination.

**Table S1** Clinical characteristics of the qualified tissue and plasma cohort

| Sample | Tissue | | Plasma | |
| --- | --- | --- | --- | --- |
| Characteristics | Normal | CRC-Ⅳ | Normal | CRC |
| Total (n) | 53 | 47(74*) | 50 | 263 |
| Gender |  |  |  |  |
| Male | 27(51%) | 30(64%) | 30(60%) | 165(63%) |
| Female | 26(49%) | 17(36%) | 20(40%) | 98(37%) |
| Age (years) | 55（25~68） | 62（47~79） | 52（22~71） | 56（25-86） |
| ≥50 | 36(68%) | 38(81%) | 29(58%) | 208(79%) |
| ＜50 | 17(32%) | 9(19%) | 21(42%) | 55(21%) |
| Stage |  |  |  |  |
| Ⅰ | NA |  | NA | 53(20%) |
| Ⅱ | NA |  | NA | 66(25%) |
| Ⅲ | NA |  | NA | 60(23%) |
| Ⅳ | NA | 34(46%) | NA | 84(32%) |
| Hepatic  metastasis^#^ | NA | 40(54%) | NA |  |
| CEA quantification |  |  |  |  |
| CEA≥ 5ng/ml | NA | NA | 0 | 97(37%) |
| CEA< 5ng/ml | NA | NA | 50 | 166(63%) |
| Comorbidities |  |  |  |  |
| Cardiovascular disease | NA | 26(55%) | 24(48%) | 139(53%) |
| Diabetes | NA | 10(21%) | 8(16%) | 47(18%) |

* Total of 74 tumor tissues from 49 stage IV CRC patients;

# Hepatic metastatic tissues from stage IV CRC patients;

** 27 paired Ⅳ stage CRC tissues and its hepatic metastasis.

**Table S2** Confusion matrices built from the ctDNA-based model in the training (A) and validation (B) cohorts.

A

| **Training cohort** | **Real stage  IV** | **Real stage  I/II** |
| --- | --- | --- |
| **Predicted  stage IV** | 43 | 3 |
| **Predicted stage I/II** | 4 | 68 |
| **Sensitivity（%）** | 91.84 |  |
| **Specificity (%)** |  | 95.78 |

B

| **Validation cohort** | **Real  stage  IV** | **Real stage  I/II** |
| --- | --- | --- |
| **Predicted  stage IV** | 28 | 6 |
| **Predicted  stage I/II** | 7 | 42 |
| **Sensitivity (%)** | 80 |  |
| **Specificity (%)** |  | 87.5 |

**Table S3** Univariate and multivariate analysis by clinicopathological variables and the ctDNA methylation model

| **Variable** | **Univariate analysis** | | | **Multivariate analysis** | | |
| --- | --- | --- | --- | --- | --- | --- |
|  | HR | (95% CI) | P | HR | (95% CI) | P |
| **Age,** ≤50(14, 23%) versus >50(46,77%) | **1.97** | 0.84-4.62 | .12 | 2.32 | 0.67-8.03 | .18 |
| **Gender,**female (22, 37%) versus male (38, 63%) | 0.95 | 0.41-2.21 | .90 | 2.47 | 0.79-7.67 | .12 |
| **T stage,** T4(34, 57%) versus T1-3(26, 43%) | **1.31** | 0.57-3.01 | .52 | 0.60 | 0.18-2.05 | .42 |
| **N stage,** N2 (21, 35%) versus N1(39, 65%) | **1.54** | 0.68-3.48 | .30 | 1.16 | 0.36-3.75 | .81 |
| **Tumor sit**e, right(11, 18%) versus left (49, 82%) | 0.92 | 0.31-2.70 | .88 | 2.30 | 0.52-10.19 | .27 |
| **Differentiation,** low(11, 19%) versus moderate-high (47, 81%) | 0.82 | 0.28-2.41 | .72 | 3.11 | 0.72-13.48 | .13 |
| **Pre-CEA,** high (22, 37%) versus normal (38, 63%) | **3.40** | 1.50-7.70 | **.003** | 0.62 | 0.17-2.26 | .46 |
| **Post-CEA,** high(9, 15%) versus normal (51, 85%) | **2.46** | 0.98-6.22 | **.057** | 0.60 | 0.17-2.07 | .41 |
| **Methylation model**, high (23, 38%) versus low (37, 62%) | **24.15** | 7.08-82.42 | 3.7e-07 | **78.94** | 14.53-428.85 | **4.21e-07** |
